# Supplementary material for: Murine and Human Gastric Tissue Establishes Organoids after 48 Hours of Cold Ischemia Time during Shipment
Source: Biomedicines. 2023 Jan 6;11(1):151. doi: 10.3390/biomedicines11010151 (PMC9855865; doi:10.3390/biomedicines11010151)
Supplement: Supplementary file 1 [file biomedicines-11-00151-s001.zip › biomedicines-2085488-supplementary.pdf]

Supplementary Material

Murine and human gastric tissue establishes organoids after 48 hours of cold ischemia time during shipment

Daniel Skubleny*, Saurabh Garg, Jim Wickware, Kieran Purich, Sunita Ghosh, Jennifer Spratlin, Dan Schiller, Gina R. Rayat

Supplemental Contents

Supplemental Methods
    Components of cold storage solutions
Table S1
Table S2
Figure S1
Figure S2
S1: Dissociation viability supplement
    Figure S3
    Figure S4
    Table S3
S2: Viability organoid assessment
    Figure S5
    Figure S6
    Figure S7
S3: Organoid growth rate assessment
    Figure S8
    Figure S9
    Figure S10
S4: In-vitro organoid dose response assays
    Figure S11
Table S4
Dataset S1

Supplemental Methods

Components of cold storage solutions

Hank's Balanced Salts

- Calcium Chloride (0.1396 g/L)
- Magnesium Sulfate (0.09767 g/L)
- Potassium Chloride (0.4 g/L)
- Potassium Phosphate Monobasic (0.06 g/L)
- Sodium Chloride (8.0 g/L)
- Sodium Phosphate Dibasic (0.04788 g/L)
- D-Glucose (1.0 g/L)
- Phenol Red-Na (0.011 g/L)

University of Wisconsin Solution (Belzer UW Cold Storage Solution)

- Osmolarity 320 milliosmoles/kilogram
- Sodium 29 miliequivalents/Liter (mEq/L)
- Potassium 125 mEq/L
- Hydroxylethyl starch (Pentafraction) 50 grams/liter
- Lactobionic acid 105 millimolar/liter (mmol/L)
- Potassium dihydrogen phosphate 25 mmol/L
- Magnesium sulfate heptahydrate 5 mmol/L
- Raffinose pentahydrate 30 mmol/L
- Adenosine 5 mmol/L
- Allopurinol 1 mmol/L
- Total Glutathione 3 mmol/L
- Potassium hydroxide 100 mmol/L

Histidine Tryptophan Ketoglutarate (Global Transplant Solutions, Servator H HTK solution)

- Osmolarity 310 miliosomoles/liter
- Sodium 15 mmol/L
- Potassium 10 mmol/L
- Magnesium 4 mmol/L
- Calcium 0.015 mmol/L
- Ketoglutarate/glutamic acid 1 mmol/L
- Histidine 198 mmol/L
- Mannitol 30 mmol/L
- Tryptophan 2 mmol/L

Table S1: Mouse organoid reagents

Reagent	Source	Catalog Number
Advanced DMEM/F12	Gibco	12634010
L-Glut	Sigma	G8540
HEPES	Fisher Scientific	BP310
Penicillin Streptomycin	Gibco	15140122
Amphotericin B	ThermoFisher	15290026
N2 Supplement	Gibco	17502011
B27 Supplement	Gibco	17504044
N-acetylcysteine	Sigma	A9165
Gastrin	Sigma	G9145
Nicotinamide	Sigma	N0636
SB202190/p 38 inhibitor	Sigma	S7067
A83-01 (ALK4/5/7 inhibitor)	Sigma	SML0788
Mouse EGF	Life Technologies	PMG8041
Human EGF	Gibco	PHG0313
Dispase II	Sigma	D4693
Collagenase IX	Sigma	C9407
Y-27632	Sigma	Y0503

Table S2: Immunofluorescence protocols

Protocol	Primary Antibody	Secondary antibody	Antigen Retrieval
1	Rabbit anti-pan cytokeratin, 1:50, overnight 4 degrees Celsius, Abcam, ab9377	Goat anti-rabbit IgG Alexa Fluor 488, 1:100, 30 minutes room temperature, Abcam, ab150077	Sodium Citrate
	Mouse anti-MUC5AC, 1:100, overnight 4 degrees Celsius, Invitrogen,	Goat anti-mouse IgG Alexa Fluor 568, 1:50, 30 minutes room temperature, Invitrogen, A-11004	
2	Rabbit anti-pan cytokeratin, 1:50, overnight 4 degrees Celsius, Abcam, ab9377	Goat anti-rabbit IgG Alexa Fluor 488, 1:100, 30 minutes room temperature, Abcam, ab150077	Sodium Citrate
	Mouse anti-TROY, 1:50, overnight 4 degrees Celsius, Santa Cruz, sc-398526	Goat anti-mouse IgG Alexa Fluor 568, 1:50, 30 minutes room temperature, Invitrogen, A-11004	
3	Rabbit anti-pan cytokeratin, 1:50, overnight 4 degrees Celsius, Abcam, ab9377	Goat anti-rabbit IgG Alexa Fluor 488, 1:100, 30 minutes room temperature, Abcam, ab150077	Sodium Citrate
	Mouse anti-LGR5, 1:50, overnight 4 degrees Celsius, Invitrogen, MA5-25644	Goat anti-mouse IgG Alexa Fluor 568, 1:50, 30 minutes room temperature, Invitrogen, A-11004	

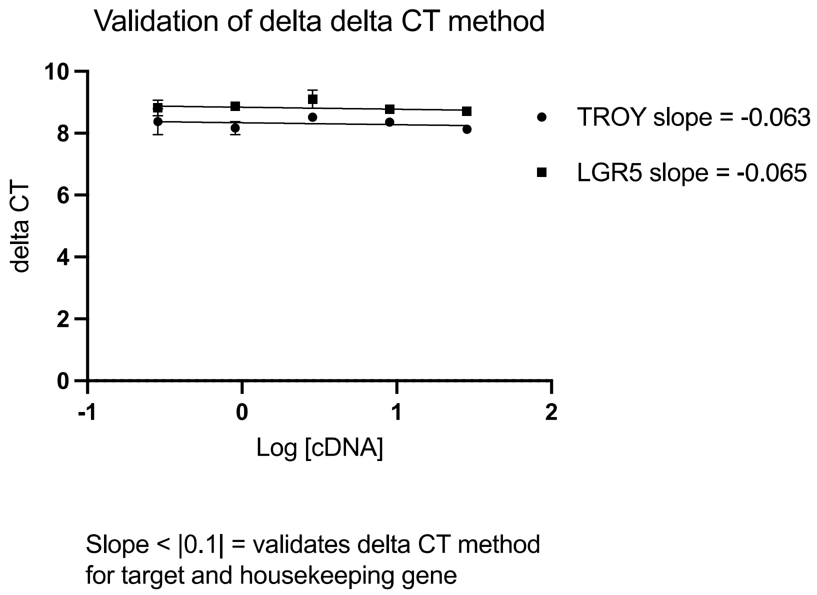

Figure S1. Delta-Delta CT validation experiment. To establish a valid experiment the efficiency of amplification for the target and reference primer must be approximately equal across multiple cDNA concentrations. Here the delta CT value versus log cDNA concentration has a slope < 0.1 indicating equal primer efficiency.

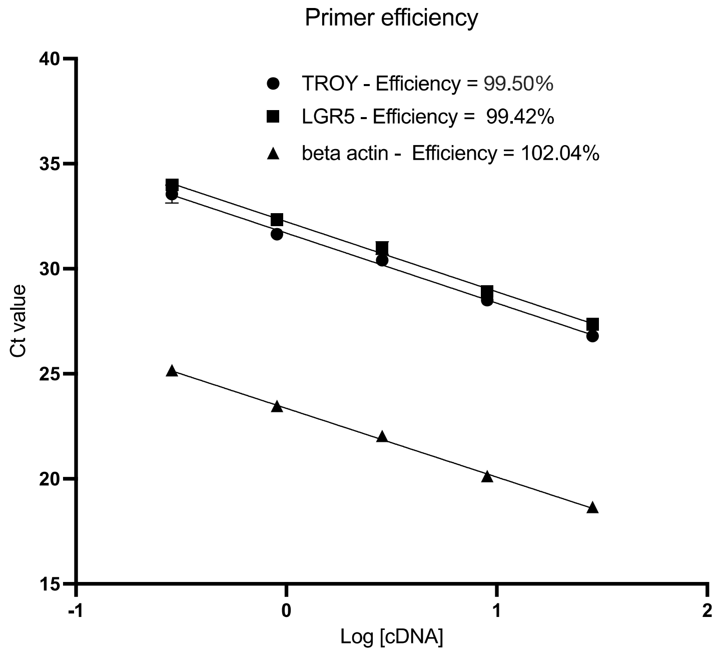

Figure S2. qRT-PCR assay primer efficiency. The efficiency is calculated as E = $(10^{-1/slope} – 1) \times 100$. All primers used in our qPCR experiments approximate 100% efficiency.

S1: Dissociation viability supplement

We assessed dissociation viability of cells prior to the first organoid plating to determine the effect of storage media and dissociation time. In this supplement we demonstrate that HBSS trended to have greater dissociation viability, albeit not significant, compared to UW and HTK solutions among fresh tissues that were dissociated within 1 hour or surgical resection (Figure S3). Of note, fresh stomachs were still stored in experimental media conditions. In Figure S4, we demonstrate that there was no significant variability in dissociation viability between Fresh, 24 hour or 48 hour dissociations for all three media conditions. In Table S3, we expand on Table 1 from the main text by showing multivariable linear regression results exclusively for tissues dissociated after 24 and 48 hours. Here, we add the temperature of the media as a variable to account for potential confounding and determine that there is no difference in dissociation viability between tissues dissociated after 24 or 48 hours of cold storage.

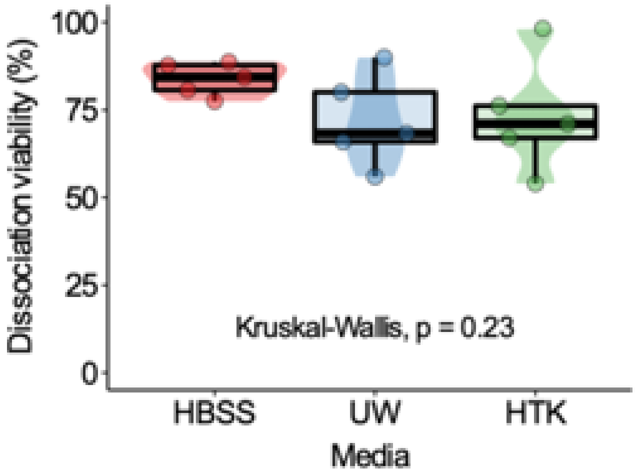

Figure S3. Dissociation viability measured by trypan blue method of fresh organoids processed immediately (within 1 hour) following surgical resection according to storage media.

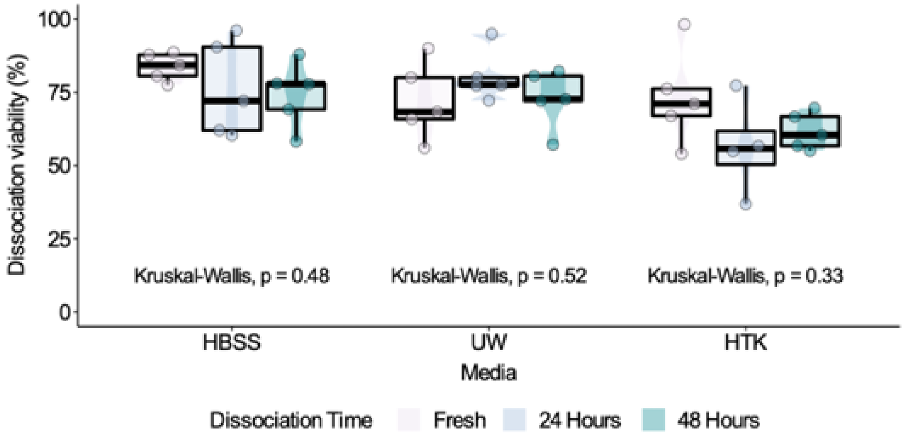

Figure S4. Dissociation viability measured by trypan blue method for all organoids according to the storage media (x-axis) and dissociation time.

Table S3: Multivariable linear regression for dissociation viability after 24- and 48-hours considering media temperature

Covariate	Beta	95% CI[1]	p-value
Media Temperature	-3.0	-6.3, 0.38	0.079
Cohort			
1	—	—	
2	-13	-27, 2.0	0.088
3	-30	-59, -2.0	**0.037**
4	-9.3	-20, 1.5	0.086
5	-20	-35, -4.9	**0.012**
Age of Mouse	-0.22	-0.51, 0.08	0.14
Media			
HBSS	—	—	
UW	1.3	-6.3, 8.9	0.7
HTK	-14	-23, -4.3	**0.006**
Dissociation Time			
24 Hours	—	—	
48 Hours	10	-4.5, 25	0.2

[1]CI = Confidence Interval

S2: Viability organoid assessment

Unadjusted viability

We analyzed the viability of mouse gastric organoids to determine the effect of storage media and dissociation time. In Figure S-1-10, we observe the trend of all viability measurements over 10 passages in each cohort. Visually, the cohort variable can be identified as a source of heterogeneity in viability and no obvious pattern relates passage to viability. Thus, despite using consistent protocols and inbred mouse stomachs, the trend of viability in gastric organoids is not consistent or reproducible. In Figure S-1-11, we demonstrate that viability is relatively unaffected by treatment media or dissociation time.

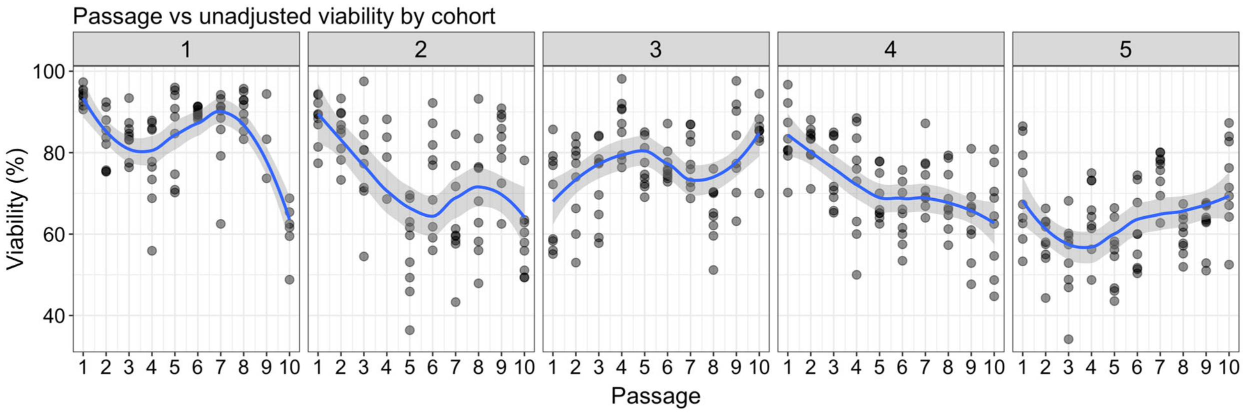

Figure S5. Unadjusted organoid viability vs passage number stratified by cohort. The y-axis is the viability measured by hemocytometer and trypan blue. The blue loess smooth is bound by the standard error in gray.

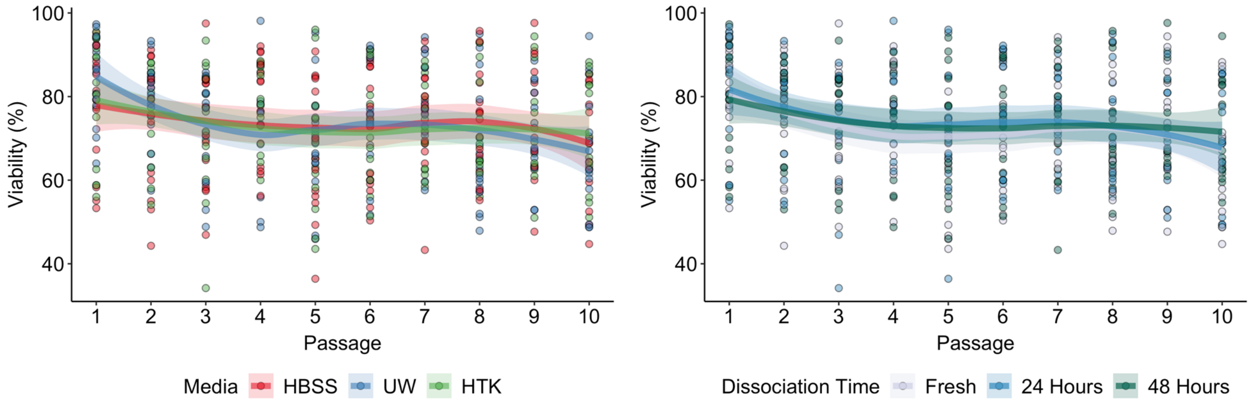

Figure S6. Unadjusted viability versus passage number by treatment media (left) and dissociation time (right). The loess smooth is bound by the standard error for each variable factor level and coloured according to the plot legend. Note, the y-axis only spans from 40-100% viability.

Multivariable models of viability over time

To determine the effect of various confounders to estimating viability in organoid culture we constructed a series of multivariable models. First, we fit multiple linear regression models with treatment media, dissociation time, number of days in culture, passage number and cohort number as variables. After examining the data visually, we observed a nonlinear relationship between viability our continuous variables. Next, we fit linear models with natural cubic splines for passage number, days in culture and growth rate. Optimal spline degrees of freedom were determined using a likelihood ratio test of nested models and a full model with the optimal spline terms was fit. The model with spline terms was superior to the first model above with an improvement in adjusted R^2 from 0.42 to 0.5. Finally, we fit generalized additive models (GAM) with penalized cubic regression splines. The GAM model was statistically superior to the spline model (Adjusted R^2 = 0.5 to 0.56). We also assessed collinearity of the cubic spline variables

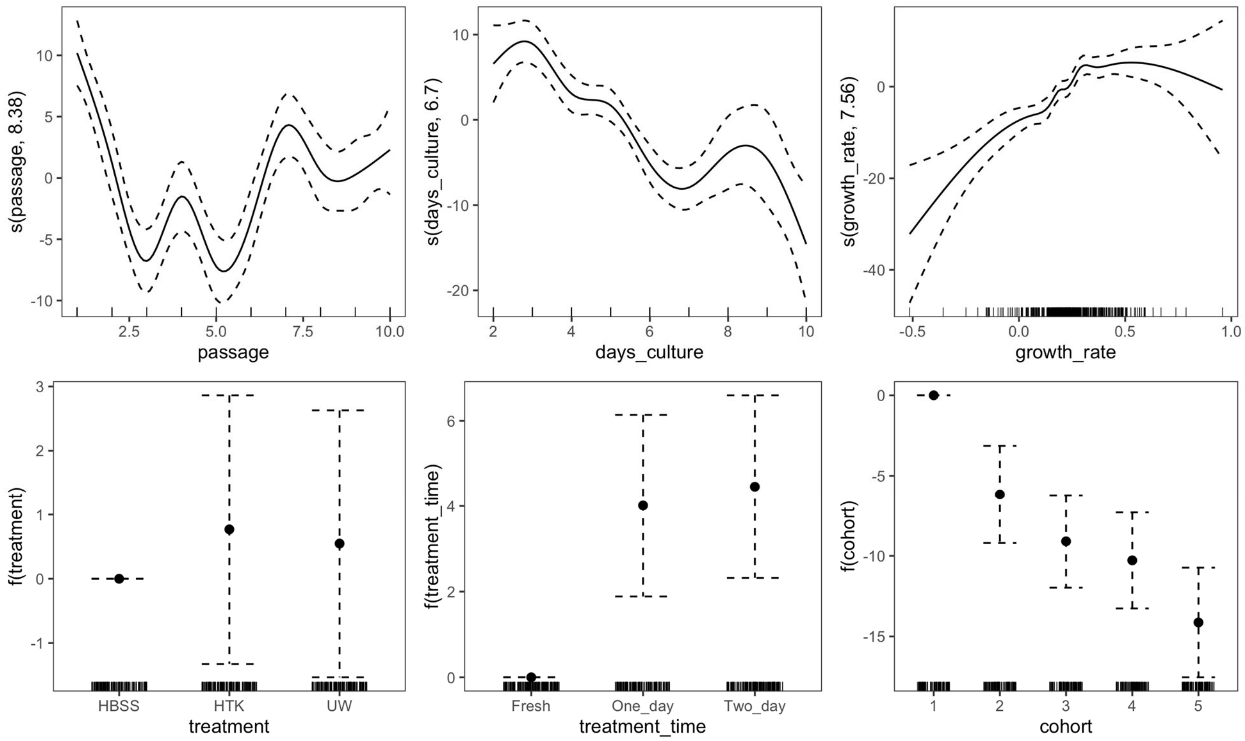

with the variable inflation factor (VIF) (Note: GAM is unable to be assessed with variable inflation factor).

Figure S7. Covariable plots from the viability generalized additive model. The top three plots show the functional effect of passage, days in culture and growth rate on viability. The bottom three images show the coefficient estimates for media, dissociation time and cohort in the context of predicting viability.

S2: Organoid growth rate assessment

<u>Unadjusted growth rate</u>

We assessed growth rate in a similar manner to our assessment of viability above.

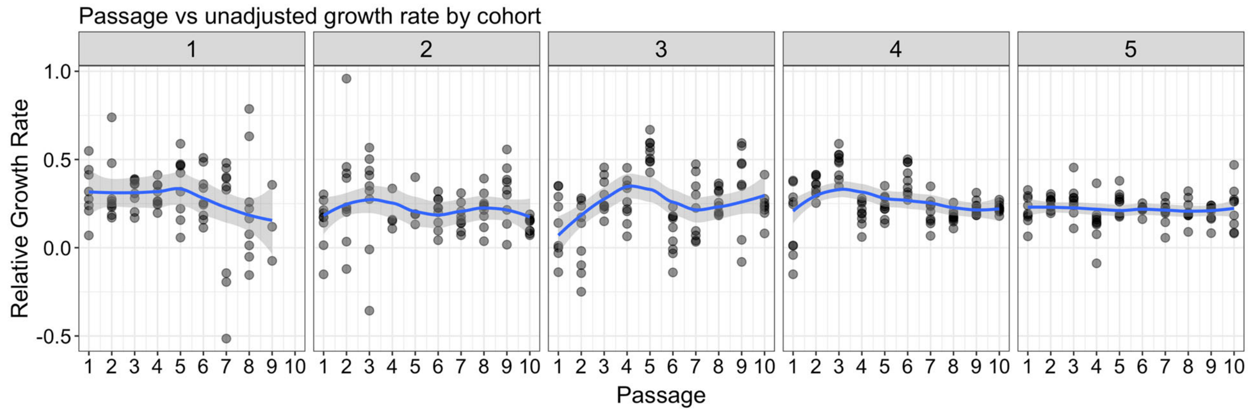

Figure S8. Unadjusted organoid growth rate vs passage number stratified by cohort. The y-axis is the growth rate measured by hemocytometer and trypan blue. The blue loess smooth is bound by the standard error in gray.

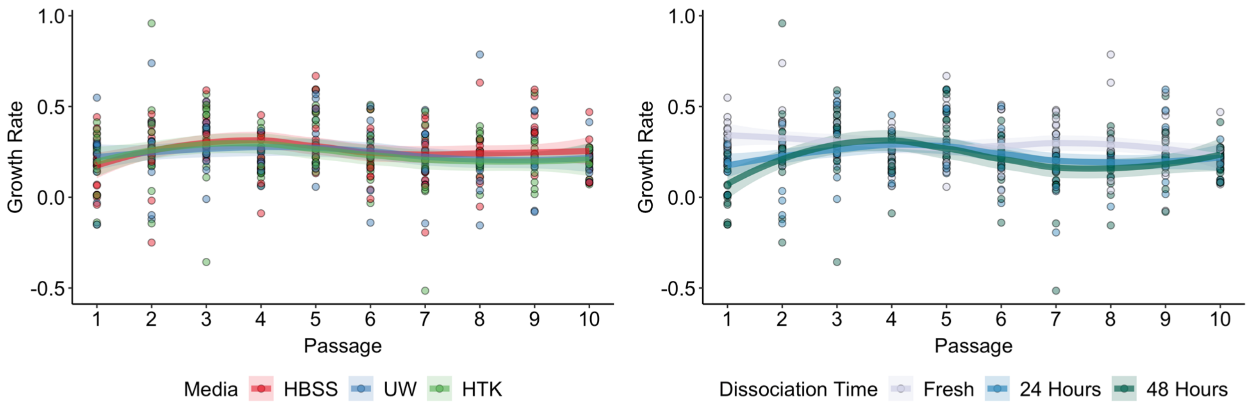

Figure S9. Unadjusted growth rate versus passage number by treatment media (left) and dissociation time (right). The loess smooth is bound by the standard error for each variable factor level and coloured according to the plot legend.

Multivariable models of relative growth rate over time

Similar to our analysis for viability above we performed a multivariable GAM regression to model the relationship of our experimental and confounding variables with growth rate.

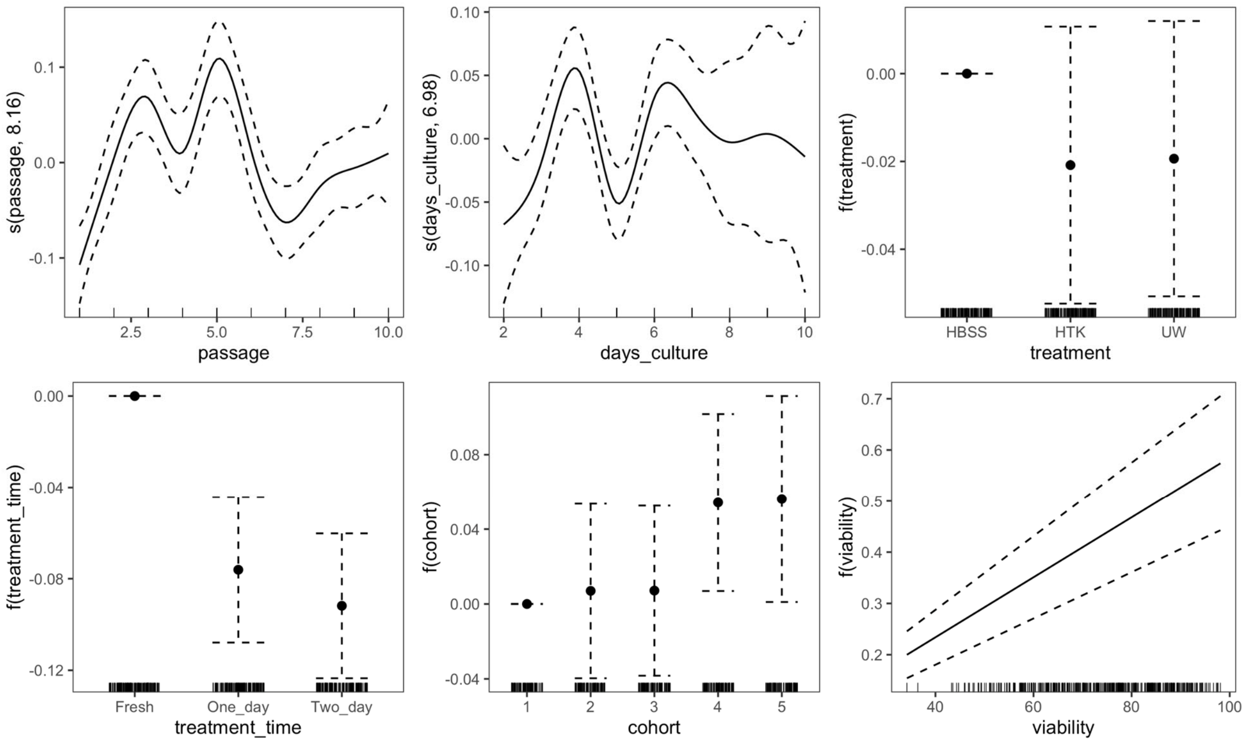

Figure S10. Covariable plots from the growth rate generalized additive model. The top three plots show the functional effect of passage, days in culture and storage media on growth rate. The bottom three images show the coefficient estimates for dissociation time, cohort and viability in the context of predicting growth rate.

S3: In-vitro organoid dose response assays

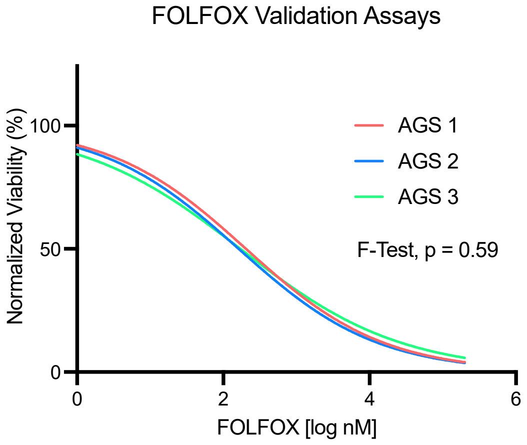

Figure S11. FOLFOX *in-vitro* drug assay validation with AGS cell line. Three independent dose-response assays were performed. Cell viability was assessed using a CCK-8 assay. Variable slope least squares linear regression was used to calculate dose-response curves. A sum-of-squares F-Test demonstrated that the was best characterized by a single dose-response curve.

Table S4. Human gastric cancer organoids overview.

Sample ID	Dissociation Time	Group	Viability	Established
53	24	24/48	79.6	yes
54	48	24/48	76.9	yes
55	Fresh	Fresh	89.2	yes
57	Fresh	Fresh	77.5	yes
59	Fresh	Fresh	96.3	yes
61	Fresh	Fresh	85.2	no
62	Fresh	Fresh	100.0	yes
63	Fresh	Fresh	98.6	yes
64	Fresh	Fresh	100.0	yes
64-24	24	24/48	53.8	no
65-Fresh	Fresh	Fresh	82.6	no
65-24	24	24/48	93.4	no
65-48	48	24/48	46.3	no
66	Fresh	Fresh	93.6	yes
67	Fresh	Fresh	84.5	yes
68-Fresh	Fresh	Fresh		no
68-24	24	24/48		no
68-48	48	24/48		no
69	Fresh	Fresh	100.0	yes
71	Fresh	Fresh	85.3	yes
72	Fresh	Fresh	96.2	yes
76	24	24/48	92.3	yes
77	Fresh	Fresh	100.0	yes
78	24	24/48	97.0	yes
79	Fresh	Fresh	75.7	yes
80	24	24/48	86.7	yes
81	Fresh	Fresh	91.1	yes
84	24	24/48	93.7	yes

Dataset S1. Data used for all analyses. Available at: https://github.com/skubleny/mouse-organoid
